# Adaptation and reliability of the Readiness for Inter professional Learning Scale in a Danish student and health professional setting

**DOI:** 10.1186/s12909-016-0591-7

**Published:** 2016-02-16

**Authors:** Birgitte Nørgaard, Eva Draborg, Jan Sørensen

**Affiliations:** Institute of Public Health, University of Southern Denmark, J.B. Winsløws Vej 9B, 5000 Odense C, Denmark

## Abstract

**Background:**

Shared learning activities aim to enhance the collaborative skills of health students and professionals in relation to both colleagues and patients. The Readiness for Interprofessional Learning Scale is used to assess such skills. The aim of this study was to validate a Danish four-subscale version of the RIPLS in a sample of 370 health-care students and 200 health professionals.

**Methods:**

The questionnaire was translated following a two-step process, including forward and backward translations, and a pilot test. A test of internal consistency and a test–retest of reliability were performed using a web-based questionnaire.

**Results:**

The questionnaire was completed by 370 health care students and 200 health professionals (test) whereas the retest was completed by 203 health professionals. A full data set of first-time responses was generated from the 570 students and professionals at baseline (test).

Good internal association was found between items in Positive Professional Identity (Q13–Q16), with factor loadings between 0.61 and 0.72.

The confirmatory factor analyses revealed 11 items with factor loadings above 0.50, 18 below 0.50, and no items below 0.20. Weighted kappa values were between 0.20 and 0.40, 16 items with values between 0.40 and 0.60, and six items between 0.60 and 0.80; all showing p-values below 0.001.

**Conclusion:**

Strong internal consistency was found for both populations. The Danish RIPLS proved a stable and reliable instrument for the Teamwork and Collaboration, Negative Professional Identity, and Positive Professional Identity subscales, while the Roles and Responsibility subscale showed some limitations. The reason behind these limitations is unclear.

## Background

Shared-learning activities have recently entered the health-care curriculum to prepare students for collaboration with colleagues and with patients. Interprofessional learning primarily aims to reduce prejudice among professionals, improve awareness of the roles and duties of other professional groups, and advance teamwork and collaborative competencies. The new activities in the education of health-care students have raised the problem of measuring the effects on student attitudes, a challenge which prompted Parsell and Bligh [1] to develop the Readiness for Interprofessional Learning Scale (RIPLS). The first version of the questionnaire had 19 items, with three subscales relating to Teamwork and Collaboration, Professional Identity, and Roles and Responsibilities. The instrument was further developed by McFadyen et al. who recommended splitting the Professional Identity subscale into Negative Professional Identity and Positive Professional Identity [2, 3]. To strengthen the third subscale, Roles and Responsibilities -while seeking new factors, such as patient-centredness, and further validating the RIPLS instrument for use with health professionals—the tool was extended to 29 items [4]. The RIPLS has been translated and adapted to fit other cultures, and by now, a Swedish [5] and Japanese version exists [6]. The translation of the instrument into Swedish and Japanese both followed a forward and backward translation process by translators being experts in both the source and the target language in order to provide a culturally relevant translation. A modified version of the English instrument with 29 items and three subscales is used in the United Arab Emirates [7]. All three versions are aimed at health-care students.

The instrument was originally developed for health-care students, and surveys have primarily involved students of health, social, and human care professions [1, 3, 5–13]. Although Reid et al. used a modified instrument aimed at health professionals [4], further research is needed to strengthen the validity and reliability of the results obtained in professional contexts.

### Danish translation

The Danish version used in our surveys of students and professionals is a translation of the original English RIPLS. De Vet, Terwee, Mokkink, and Knol’s recommendations were followed [14]. The process involved 1) forward translation from English into Danish by two native speakers of Danish working independently of each other; 2) synthesis of these two translations; 3) two independent backward translations from Danish into English by two native speakers of English; 4) review and reconciliation of the backward translations; and 5) pilot-testing on a convenience sample of 15 students, after which the final editing took place (A detailed description of the translation process is available on request from the corresponding author). To adapt the student version for use with our health professional group, the following changes were made. We replaced the Danish equivalents of *student* by *professional*, *interdisciplinary* by *interprofessional,* and the phrase *working in an interprofessional team* replaced terms describing the effect of interdisciplinary training.

The aim of this study was to validate the Danish version of the Readiness for Interprofessional Learning Scale in a sample of health-care students and professionals.

## Methods

Our surveys included a test of internal consistency and a test–retest of reliability, but only the professional group was retested. The questionnaires were administrated through the web.

Validation of the instrument aimed to check completeness of the data; sub-scale assumptions; item-discriminant validity and scaling success rates (i.e., the measure of item correlation with its proposed scale), internal consistency reliability for each subscale, test-retest reliability (for the professional sample only), and subscale precision and applicability to the specific population.

Stata Release 13 was used for all statistical analysis (StataCorp 2013. Stata Statistical Software Release 13, College Station, TX: StataCorp LP).

### Data Collection, Study Population, and Validation

The health professional group included medical doctors, registered nurses, nursing assistants, medical secretaries, and physiotherapists who participated in an intervention concerning interprofessional learning and collaboration. The data were collected in August and September of 2012 using a web-based questionnaire system. The participants received an e-mailed invitation and a link to the form. For the retest, an identical questionnaire was administered after 16 days. For each test, a reminder was dispatched 2 weeks later to those who had not submitted a response.

The Danish student version was validated in a group of health-care students of nursing, medicine, physiotherapy, occupational therapy, laboratory technology, and radiography, who participated in a study to evaluate structured interprofessional clinical training in a hospital setting. Data collection took place from August 2008 to February 2010. A web-based questionnaire was administered through an e-mail link. A reminder was sent if no response was received within 7 days. E-mail addresses were obtained from the included educational institutions.

### The Readiness for Interprofessional Learning Scale

The use of subscales in RIPLS studies has varied. The original three-subscale version has been tested in several studies [2, 8], where internal consistency tests and content analyses revealed structural limitations, leading McFadyen et al. to suggest a four-subscale structure [2], which has been followed up by thorough testing [11–13].

Individual items have also been revised. The original questionnaire contained 19 items, but was expanded for use with professional groups [4]. The resulting 29 items assessed Teamwork and Collaboration (in nine items), Negative Professional Identity (three items), Positive Professional Identity (four items), and Roles and Responsibilities (13 items). Assessments are given on a five-point Likert scale, and agreement/non-agreement on a bipolar symmetric scale (*Strongly agree, Agree, Undecided, Disagree, Strongly disagree*).

### Analyses

To ensure that no items were left unanswered, both survey systems were constructed so as to disallow blank response spaces. However, some respondents disconnected without finishing the questionnaire, and we report the proportions of both completed and partly completed responses. As the relevant information was unavailable, no analysis on non-responders was possible. Missing data are reported on the respondent level rather than on the item level.

Previous studies [1, 4, 11, 15] have indicated that four subscales are appropriate for RIPLS studies in other languages. This was evaluated for our study using a confirmatory factor analysis based on item correlations. We were prepared to reconsider the number of subscales if the association to the latent factor were found to be lower than 0.2 (<0.2). Coefficients above 0.9 (>0.9) were considered highly satisfactory [14].

Inter-discriminant validity was tested to determine the correlation between the items and the subscale assumed.

Within all subscales, Cronbach’s alpha reliability coefficients were calculated to test for internal consistency reliability. Values between 0.70 and 0.90 were taken as indicating acceptable reliability [16]. Furthermore, subscales mean scores were examined for differences between the two samples, indicating applicability issues related to certain groups.

Test–retest data (for the health professionals only) were analysed by calculating weighted kappa coefficients on each item using quadratic weights (test and retest samples). Values below 0.2 indicate poor agreement, 0.2–0.4 fair agreement, 0.4–0.6 moderate agreement, and 0.6–0.8 substantial agreement. Coefficients between 0.8 and 1.0 express perfect agreement [17].

Occasionally, questionnaires suffer from floor or ceiling effects when applied in new circumstances. As the original 19-item questionnaire was created for a student population, and later expanded to 29 items and adjusted for a population of professionals, we evaluated floor and ceiling effects by skewness of score. A high skewness of scores would indicate low precision or low applicability to the specific population.

### Ethical considerations

All the included participants are of age (18 years or older) and the return of a questionnaire, whether fully or only partially completed, was considered to express voluntary consent to participation. All personal identifiers are removed or disguised during the analyses to preclude personal identification.

The study was licensed by the Danish Data Protection Agency and needed no further ethical approval according to Danish legislation.

## Results

The questionnaire was completed by 370 students of various health-care professions, 80.5 % of whom were female (response rate 57,5 %). The proportions of students were as follows: 31.9 % nursing (registered), 13.8 % medical students, 19.2 % physiotherapy, 14.6 % occupational therapy, 14.3 % radiography, and 6.2 % medical laboratory technology.

Two hundred health professionals completed the first questionnaire (test) (response rate 41 %); the second questionnaire (retest) was completed by 203. Both forms were returned by 129 professionals. Table 1 gives numbers of respondents by age, gender, and profession. The responses by item and subscale are shown in Table 2.

**Table 1:** Students, health professionals (baseline and test–retest sample, respectively), and full data set, by age, gender, and profession

	Health-care students		Health professionals baseline		Health professionals test–retest		Full data set	
	n (370)	%	n (200)	%	n (129)	%	n (570)	%
Age								
20–29	284	52.3 %	14	7.0 %	8	6.2 %	298	52.3 %
30–39	34	6.26 %	52	26.0 %	29	22.5 %	86	15.1 %
40–49	12	2.21 %	74	37.0 %	50	38.8 %	86	15.1 %
50–59			53	26.5 %	39	30.2 %	53	9.3 %
60+			7	3.5 %	3	2.3 %	7	1.2 %
Unknown	40	10.8 %					40	7.0 %
Female gender	298	80.5 %	179	89.5 %	112	86.8 %	477	83.7 %
Profession/Study								
Medical doctor	51	13.8 %	34	17.0 %	26	20.2 %	85	14.9 %
Registered nurse	118	31.9 %	121	60.5 %	74	57.4 %	239	41.9 %
Nursing assistant			14	7.0 %	8	6.2 %	14	2.5 %
Medical secretary			26	13.0 %	18	14.0 %	26	4.6 %
Physiotherapist	71	19.2 %	1	0.5 %			72	12.6 %
Occup. therapist	54	14.6 %					54	9.5 %
Radiograph	53	14.3 %					53	9.3 %
Med.lab.tech.	23	6.2 %					23	4.0 %
Other			4	2.0 %	3	2.3 %	4	0.7 %

**Table 2:** Responses by item and subscale - full data set (n = 570)

Scale	Item	Question	Strongly disagree (%)	Disagree (%)	Neither agree, nor disagree (%)	Agree (%)	Strongly agree (%)
Teamwork and Collaboration Items 1–9 / 9 items	Q1	Learning with other students will help me become a more effective member of a health-care team	1.8	2.0	12.6	46.0	37.6
	Q2	Patients would ultimately benefit if health-care students worked together to solve patient problems	2.0	0.9	9.9	41.6	45.6
	Q3	Shared learning with other health-care students will increase my ability to understand clinical problems	2.0	2.4	13.7	47.1	34.8
	Q4	Learning with health-care students before qualification would improve relationships after qualification	1.8	2.2	15.6	47.3	33.2
	Q5	Communication skills should be learned with other health-care students	3.3	6.6	29.5	36.6	24.0
	Q6	Shared learning will help me to think positively about other professionals	1.7	4.2	22.5	46.2	25.5
	Q7	For small-group learning to work, students need to trust and respect each other	1.1	0.2	2.0	41.2	55.5
	Q8	Teamworking skills are essential for all health-care students to learn	1.3	0.6	5.1	47.1	46.0
	Q9	Shared learning will help me to understand my own limitations	2.8	2.9	25.3	45.2	23.8
Negative Professional	Q10	I don’t want to waste my time learning with other health-care students	37.9	39.9	17.0	2.0	3.1
Identity	Q11	It is not necessary for undergraduate health-care students to learn together	30.4	40.3	22.0	5.7	1.7
Items 10–12 / 3 items	Q12	Clinical problem-solving skills should only be learned with students from my own department	26.9	46.3	20.9	4.4	1.5
Positive Professional Identity Items 13–16 / 4 items	Q13	Shared learning with other health-care students will help me to communicate better with patients and other professionals	2.0	4.6	26.1	46.8	20.6
	Q14	I would welcome the opportunity to work on small-group projects with other health-care students	3.9	4.0	16.2	48.4	27.5
	Q15	Shared learning will help to clarify the nature of patient problems	1.5	2.4	24.6	48.4	23.1
	Q16	Shared learning before qualification will help me become a better teamworker	1.1	4.6	21.9	45.9	27.0
Roles and Responsibilities	Q17	The function of nurses and therapists is mainly to provide support for doctors	24.6	37.6	24.6	10.3	2.9
Items 17–29 / 13 items	Q18	I’m not sure what my professional role will be	31.6	38.5	17.3	8.4	4.2
	Q19	I have to acquire much more knowledge and skills than other health-care students	10.3	30.3	38.2	16.9	4.4
	Q20	There is little overlap between my future role and that of other health-care professionals	6.8	36.2	46.2	9.0	1.8
	Q21	I would feel uncomfortable if another health-care student knew more about a topic than I did	7.3	23.9	22.8	29.2	16.9
	Q22	I will be able to use my own judgment a lot in my professional role	0.7	2.4	16.2	63.1	17.6
	Q23	Reaching a diagnosis will be the main function of my role	8.8	29.9	34.9	19.6	6.8
	Q24	My main responsibility as a professional will be to treat my patient	3.3	5.7	13.4	49.7	27.9
	Q25	I like to understand the patient’s side of the problem	0.9	0.9	6.8	54.5	36.9
	Q26	Establishing trust with my patients is important to me	0.9	0.4	2.0	28.8	67.9
	Q27	I try to communicate compassion to my patients	0.7	0.9	3.7	36.9	57.8
	Q28	Thinking about the patient as a person is important in getting treatment right	0.7	0.9	3.7	35.1	59.6
	Q29	In my profession you need skills in interacting and cooperating with patients	1.1	0.6	3.9	32.8	61.6

In order to test the overall applicability of the questionnaire, a full data set of first-time responses was generated from the 570 students and professionals at baseline.

### Sub-scales Assumptions

Factor analyses were conducted at baseline on the full data set of students and professionals. Association was established for all items in Teamwork and Collaboration (Q1–Q9), with factor loadings between 0.46 and 0.74. Items Q5, Q6, and Q9 were also associated with Q13–Q16 in Positive Professional Identity, with factor loadings of 0.43, 0.46, and 0.49, respectively.

While the items in Negative Professional Identity (Q10–Q12) associated with the latent factor (with factor loadings between 0.40 and 0.46), they were negatively associated with Positive Professional Identity (factor loadings between −0.51 and −0.55).

Good internal association was found between items in Positive Professional Identity (Q13–Q16), with factor loadings between 0.61 and 0.72.

The Roles and Responsibilities items (Q17–Q21) showed irregular internal correlation; five items (Q17–Q21) had factor loadings between 0.26 and 0.52, while six items (Q24–Q29) were between 0.40 and 0.87. The remaining items (Q22 and Q23) were associated with Teamwork and Collaboration (factor loading 0.27) and with Negative Professional Identity (factor loading 0.43).

The uniqueness (i.e., variability) of the items ranged between 0.22 and 0.93, with 11 items above 0.50, 18 below 0.50, and no items below 0.20. The results of factor analysis are shown in Table 3.

**Table 3:** Rotated factor loadings and unique variances, by item (n = 545)

Item	Factor 1	Factor 2	Factor 3	Factor 4	Uniqueness
Q1	0.70				0.29
Q2	0.74				0.35
Q3	0.71				0.32
Q4	0.69				0.39
Q5	0.51	0.43			0.55
Q6	0.54	0.46			0.47
Q7	0.65				0.44
Q8	0.61				0.50
Q9	0.46	0.49			0.52
Q10		−0.55	0.60		0.27
Q11		−0.51	0.49		0.41
Q12		−0.51	0.40		0.50
Q13		0.70			0.41
Q14		0.62			0.42
Q15		0.61			0.48
Q16		0.72			0.36
Q17				0.26	0.88
Q18				0.52	0.72
Q19				0.46	0.67
Q20				0.29	0.90
Q21				0.30	0.89
Q22	0.27				0.85
Q23			0.43		0.78
Q24		0.40			0.81
Q25		0.75			0.41
Q26		0.87			0.21
Q27		0.73			0.42
Q28		0.75			0.36
Q29		0.83			0.26

All loadings above 0.4 are shown (Note: for all items, the largest value is always displayed)

The rotated factor loadings seem to suggest the need for an independent subscale for the last six items. To further test this hypothesis, an additional factor analysis assuming five subscales was performed. However, this analysis produced only marginally different uniqueness values; the introduction of a fifth subscale was therefore rejected (data not shown).

### Item-Discriminant Validity, Scaling Success Rates, and Internal Consistency

The item-discriminant validity of the RIPLS was tested through scaling success rates for each subscale. To test internal consistency between items, Cronbach alpha values were calculated for each subscale, with the full data set.

Table 4 shows the results for item-internal consistency, item-discriminant validity, scaling success rates, homogeneity, and reliability.

**Table 4:** Item scaling tests and reliability estimates for RIPLS subscales

		Range of item correlations		Item scaling tests		Scale	
	n items^a^	Item-internal consistency^b^	Item-discrimi-nant validity^c^	Success/Total^d^	Scaling suc-cess rate%	Homo-geneity^e^	Relia-bility^f^
Teamwork and Collaboration	9	0.70–0.83	0.12–0.58	36/36	100	0.64	0.91
Negative Professional Identity	3	0.83–0.90	0.00–0.69	9/12	75	0.81	0.84
Positive Professional Identity	4	0.80–0.85	0.01–0.59	16/16	100	0.74	0.84
Roles and Responsibility	13	0.17–0.69	0.01–0.38	44/52	85	0.37	0.61

^a^Number of items in subscales and number of item-internal consistency tests per scale

^b^Correlations between items and scale

^c^Correlations between items and other scales

^d^Number of significantly higher/total number of correlations

^e^Average inter-item covariance (SD)

^f^Internal-consistency reliability (Cronbach alpha)

### Test-Retest

Item responses were coded as follows: *Strongly disagree* = 5; *Disagree* = 4; *N*e*ither agree nor disagree* = 3; *Agree* = 2; *Strongly agree* = 1. Mean scores were calculated by item, and stability over time was found, with 11 items showing identical mean scores in both test and retest samples; 11 items saw a 0.1 numerical decrease in test–retest mean scores; four items decreased by 0.2, while four items saw a 0.1 increase. The mean values in the test-to-retest scores thus changed only marginally (data not shown).

Weighted kappa analysis was performed in order to test for reproducibility, resulting in seven items with kappa values between 0.20 and 0.40, 16 items with values between 0.40 and 0.60, and six items between 0.60 and 0.80; all showing *p*-values below 0.001. Table 5 displays all kappa scores.

**Table 5:** Weighted kappa values (professionals, test-retest sample, n = 129)

Item	Kappa (Wt)
Q1	0.42
Q2	0.37
Q3	0.41
Q4	0.40
Q5	0.50
Q6	0.48
Q7	0.44
Q8	0.47
Q9	0.33
Q10	0.51
Q11	0.56
Q12	0.63
Q13	0.70
Q14	0.60
Q15	0.63
Q16	0.62
Q17	0.59
Q18	0.41
Q19	0.51
Q20	0.27
Q21	0.54
Q22	0.33
Q23	0.61
Q24	0.37
Q25	0.33
Q26	0.45
Q27	0.47
Q28	0.39
Q29	0.49

### Subscale Precision and Applicability

For each subscale, the proportions of minimum and/or maximum scores among students and professionals were calculated. These counts indicate whether the respondents tended to cluster in the highest or the lowest score categories. In the three subscales with positively formulated items, mean scores were above 3.46 for all samples, and equal to or above 4.0 in two of the three scales. In the subscale with negatively formulated items (Table 2, Q10–12), the mean was 2.0 for each sample, thus indicating skewness, as shown in Table 6.

**Table 6:** Descriptive statistics of score distributions for subscales, by sample

	Students	Professionals (baseline)	Professionals (test)	Full data set
	Mean (SD) skewness	Mean (SD) skewness	Mean (SD) skewness	Mean (SD) skewness
Teamwork and Collaboration	4.0 (0.60)-1.1	4.2 (0.69)-2.0	4.2 (0.77)-2.2	4.1 (0.64)-1.4
Negative Professional Identity	2.1 (0.86) 0.8	1.9 (0.72) 0.3	1.8 (0.65) 0.4	2.0 (0.81) 0.8
Positive Professional Identity	3.8 (0.74)-0.7	4.0 (0.73)-0.7	4.1 (0.78) .-1.0	3.9 (0.74)-0.7
Roles and Responsibility	3.6 (0.38)-1.6	3.5 (0.3)-0.3	3.5 (0.35)-0.5	3.6 (0.37)-1.2

The entire data set was tested for floor and ceiling effects. For Teamwork and Collaboration, between 23 % and 55 % of respondents gave the top rating, *Totally agree*, whereas 1–3 % gave the bottom rating, *Totally Disagree*. For Negative Professional Identity, top ratings were given by 1–3 % and bottom rating by 26–37 %. For the items in Positive Professional Identity, 20–27 % gave top ratings and 1–3 % bottom rating. The first three items in Roles and Responsibility (Q17–Q19) attracted top ratings from 2–4 % and bottom from 10-31 %; for Q20, the proportions were 1 % giving top ratings, and 6 % giving bottom rating. Items Q21–Q23 were top-rated by 6–17 % and bottom-rated by 0.7-8 % of the respondents. The remaining six Roles and Responsibility items (Q24–Q29) showed 27–67 % top ratings and 0.7–3 % bottom ratings (Table 2).

## Discussion

This study has demonstrated that three of the four subscales in the Danish version of the RIPLS instrument contribute to it being an appropriate tool for assessing attitudes towards shared learning among students and professionals in the health services. The analysis showed strong internal consistency within the three subscales of Teamwork and Collaboration, Negative Professional Identity, and Positive Professional Identity, with factor loading and alpha values above the recommended thresholds. However, the Roles and Responsibility subscale showed some limitations in the Danish setting, with a low alpha (0.61) and loadings exceeding the criterion thresholds.

The analysis of test-retest reliability revealed that the instrument is stable and reproducible, with weighted kappa scores lying between 0.27 and 0.70. This indicates fair to substantial agreement with the lowest values in Roles and Responsibility. While these results suggest a need for a fifth subscale - as proposed by [18] - the factor analysis did not support this assumption. Other researchers have suggested creating a new subscale from items 25–29 to assess Patient-centredness [4, 7]. However, in our factor analysis, item 24 showed correlation with Items 25–29. Besides, we also found a correlation between items 17–22 assessing Roles and Responsibilities, thus partly matching the results reported in previous research using three subscales [1, 2], or with reduced scales of 23 items [4] or 20 items [7].

The RIPLS instrument was originally developed for English students, and later proved stable and reliable for use with professionals (1–3). In our 29-item version with four subscales, which we used with both student and professional populations, factor loadings were above 0.40 for all but three items, and with an overall alpha of 0.89, internal consistency was good. Our results support those of the aforementioned Swedish study, while the comparison with the Japanese study (both 19 items, 4 subscales) is less convincing. In our study, both Cronbach alpha and the majority of the factor loadings were above the recommended thresholds [14, 16].

Regarding test-retest reliability, no kappa values below 0.2 were identified. We found seven values between 0.2 and 0.4, indicating fair agreement; 16 kappa values were between 0.4 and 0.6, indicating moderate agreement, and six results lay between 0.6 and 0.8, indicating substantial agreement. Relating these results to the four subscales, the strongest agreement was found with Positive Professional Identity (all values above 0.6), followed by Negative Professional Identity (two values between 0.4 and 0.6, one above 0.6). The results for the remaining subscales are more ambiguous, with Teamwork and Collaboration (nine items) showing two values below the 0.2 threshold and seven values between 0.4 and 0.6. For the 13 items in Roles and Responsibilities, we found five values between 0.2 and 0.4, seven between 0.4 and 0.6, and one above 0.6. A review of the literature revealed only a single study that assessed the test-retest reliability of RIPLS: McFadyen, Webster, and Maclaren (2006) used a weighted kappa, with 19 items tested on 65 professionals. With the exception of Roles and Responsibilities (which had only three items), its subscales were identical to those used by us. McFadyen et al. presented weighted kappa values between 0.12 (Item 12) and 0.55 (Item 1), being lowest for Positive Professional Identity (0.12–0.39) and Negative Professional Identity (0.31–0.55; [19]. These results differ considerably from ours, where the mentioned subscales showed the highest kappa results.

Only minor differences appeared between test results for the student and the professional setting. Comparing the subscale results for the two cohorts, we found students’ score means to be significantly higher only for Roles and Responsibilities. Their Cronbach alpha scores were also better than the professionals’- although only negligibly so.

The full data set was also tested for ceiling and floor effects, which are defined as situations in which at least 15 % of responses give the highest or lowest possible score [20]. A ceiling effect was found for all items in Teamwork and Collaboration, Positive Professional Identity, and Negative Professional Identity; the situation was more ambiguous with Roles and Responsibilities, where there was a ceiling effect for Items 21–22 and 24–29 and a floor effect for Items 17–18. No earlier research has presented results indicating floor or ceiling effects, which could reflect the fact that, with only five response categories, each answer will attract on average 20 % of responses, thus making the 15 % threshold too narrow. However, McFadyen, Webster, and Maclaren found that Roles and Responsibilities items had, in general, lower scores, leading them to reconsider the score for these items [19]. While floor or ceiling effects pose no substantial challenge to validity studies, the ceiling effects found in this study in particular may indicate problems, as they leave limited room for future intervention studies to demonstrate improvement [14]. The ceiling effect might also reflect a possible selection bias, as responders would have had relatively positive attitudes to interprofessional learning. Drop-out analyses would therefore be useful in order to prove casemix representativity.

Other studies in the field did not reveal whether questionnaires were administered by surface mail or electronic mail. All samples of this study were collected using an e-mail that provided a link to a web-based questionnaire, a method that Looij-Jansen and De Wilde have shown not to influence results or response rates [21].

In De Vet et al.’s view, the adaptation of an instrument across cultural and linguistic boundaries basically requires a translation and a test of score equivalence to ensure comparability of scores across boundaries [14]. Comparison with the Swedish study is precluded, as this used other subscales [5]; and the Japanese study reported neither individual item scores nor subscale means [6]. Claiming that cross-cultural adaptation is successful would thus be contentious; on the other hand, the concordant findings from both groups in our study indicate that the RIPLS is a stable and reliable instrument for use in a Danish setting with professionals as well as students.

## Conclusion

We have shown that the Danish version of the RIPLS is a stable and reliable instrument for use in both a student and a professional population with regard to the Teamwork and Collaboration, Negative Professional Identity, and Positive Professional Identity subscales (Items 1–16).

The remaining 13 items (17–29), used for the assessment of Roles and Responsibility, proved unsuitable as a subscale, whether with students or professionals. It remains unclear if this divergence from previous research results should be interpreted as a reflection of cultural differences. We recommend further studies in order to test for the feasibility of introducing a division of the Roles and Responsibility subscale.

## Abbreviations

Q: question
RIPLS: readiness for interprofessional learning scale

## References

[1] Parsell G, Bligh J (1999). The development of a questionnaire to assess the readiness of health care students for interprofessional learning (RIPLS). Med Educ.

[2] McFadyen AK (2005). The Readiness for Interprofessional Learning Scale: a possible more stable sub-scale model for the original version of RIPLS. J Interprof Care.

[3] McFadyen AK, Maclaren WM, Webster VS (2007). The Interdisciplinary Education Perception Scale (IEPS): an alternative remodelled sub-scale structure and its reliability. J Interprof Care.

[4] Reid R (2006). Validating the Readiness for Interprofessional Learning Scale (RIPLS) in the postgraduate context: are health care professionals ready for IPL?. Med Educ.

[5] Lauffs M (2008). Cross-cultural adaptation of the Swedish version of Readiness for Interprofessional Learning Scale (RIPLS). Med Educ.

[6] Tamura Y (2012). Cultural adatation and validating a japanese version of the readiness for interprofessional learning scale (RIPLS). Journal of Interprofessional Care.

[7] El-Zubeir M, Rizk DE, Al-Khalil RK (2006). Are senior UAE medical and nursing students ready for interprofessional learning? Validating the RIPL scale in a Middle Eastern context. J Interprof Care.

[8] Horsburgh M, Lamdin R, Williamson E (2001). Multiprofessional learning: the attitudes of medical, nursing and pharmacy students to shared learning. Med Educ.

[9] Rose MA (2009). Attituddes of Students in Medicine, Nursing, Occupational, and Physical Therapy Toward Interprofessional Education. J Allied Health.

[10] Goelen G (2006). Measuring the effect of interprofessional problem-based learning on the attitudes of undergraduate health care students. Med Educ.

[11] McFadyen AK (2010). Interprofessional attitudes and perceptions: Results from a longitudinal controlled trial of pre-registration helath and social care students in Scotland. Jorunal of Interprofessional Care.

[12] Bradley P, Cooper S, Duncan F (2009). A mixed-methods study of interprofessional learning of resuscitation skills. Med Educ.

[13] Curran VR (2010). A longitudinal study of the effect of an interprofessional education curriculum on student satisfaction and attitudes towards interprofessional teamwork and education. J Interprof Care.

[14] De Vet HCW (2011). *Measurement in Medicine*. Practical Guides to Biostatistics and Epidemiology. Vol. 1.st.

[15] McFadyen AK (2012). The readiness for Interprofessional learning Scale: A possible more stable sub-scale for the original version of RIPLS. Jorunal of Interprofessional Care.

[16] Nunnally JC, Bernstein IH (1994). *Psychometric Theory*. Vol. Third.

[17] Landis JR, Koch GG (1977). The measurement of observer agreement for categorical data. Biometrics.

[18] Cooper H, Spencer-Dawe E, McLean E (2005). Beginning the process of teamwork: Design, impementation and evaluation of an inter-professional education intervention for first year undergraduate students. Journal of Interprofessional Care.

[19] McFadyen AK, Webster VS, Maclaren WM (2006). The test-retest reliability of a revised version of the Readiness for Interprofessional Learning Scale (RIPLS). J Interprof Care.

[20] Terwee CB (2007). Quality criteria were proposed for measurements properties of health status questionnaires. J Clin Epidemiol.

[21] Looij-Jansen PM, De Wilde EJ (2008). Comparison of Web-Based versus Paper-and-Pencil Self-Administered Questionnaire: Effects on Health Indicators in Dutch Adolescents. Health Serv Res.

